# Open to All: Dementia, Creativity, and Open Ecosystem Innovation

**DOI:** 10.3389/fsoc.2019.00010

**Published:** 2019-03-15

**Authors:** Timothy J. Senior

**Affiliations:** Digital Cultures Research Centre, University of the West of England, Bristol, United Kingdom

**Keywords:** open innovation, collaboration, hubs, ecosystems, health, dementia, creativity

## Abstract

In the health arena, open innovation approaches strive to address real-world complexity through driving multi-stakeholder collaborative activities that can better identify and respond to complex health needs. This paper will argue for the value of an open ecosystem innovation approach, one that explores the full implications of what it means to be “open” in a health innovation context. To these ends, the paper will outline the origins of open innovation in the health arena, suggesting that it has become an important site for pushing the limits of open methods and challenging mainstream conceptions of the targets of health innovation. Five guiding principles for open ecosystem innovation will then be proposed, drawing on learning from the Knowledge Exchange Hubs for the Creative Economy funded by the UK's Arts and Humanities Research Council. These principles point to a configuration of open activities that are maximally sensitive to (1) knowledge diversity in innovation work; (2) the consequences of adopting an open-orientation across all stages of innovation programming; (3) the value of deepening and broadening the targets of innovation activity; (4) the role of mediation in supporting cross-sector partnerships; and, (5) the importance of operating in an adaptive and sustainable manner in the long-term. A follow-on project from the AHRC Hubs—Dementia Connect—sought to apply this learning to an important health focus: dementia and the role played by creative participation in delivering important health outcomes. Through Dementia Connect, the applicability of open ecosystem innovation thinking was assessed, revealing the conditions under which it might deliver innovation-led improvements to the quality of life for those living with a dementia diagnosis. A detailed blueprint for conducting open ecosystem innovation is then proposed in full—a new and comprehensive response to the complex reality of living with a dementia diagnosis today.

## 1. Introduction to open Innovation in Health

In the health arena, open innovation approaches strive to address real-world complexity through driving multi-stakeholder collaborative activities that can better identify and respond to complex health needs. The development of open approaches is still very much an ongoing process, with their full potential in the health arena not yet fully realised. The subject of this paper—a new model of open ecosystem innovation for the dementia and creativity arena—is one proposal for advancing that development.

### 1.1. The Emergence of Open Approaches

The emergence of open innovation models can be traced back over the last 40 years through changing attitudes to the sites, participants, and outputs of innovation-oriented work within industry: from a focus on business differentiation (with successful product and service development tied to internal research and development—R&D), through a focus on core competences (in which an R&D base is broadened through outsourcing), to an open innovation focus in which greater emphasis is placed on the acceleration of internal innovation activities and the expansion of external markets through reciprocal interactions with external partners (Chesbrough, 2003; Sargsyan et al., 2011). In this account, the coupling of “open” activities simultaneously across different businesses becomes a form of networked innovation. In essence, open approaches are grounded in a single notion: that individual organisations can no longer have a monopoly on R&D or knowledge mobilisation. In other words, “internal knowledge” can no longer be relied on in sustaining current market positions or for survival in a global market in which knowledge is highly distributed across organizations and individuals (Chesbrough et al., 2006). Notwithstanding open innovation's traditional market-orientation, it is the broader scope of open activities beyond business and private economic agents that is now gaining prominence, reflecting their considerable value in other fields of work (Amin and Cohendet, 2004; Lester and Piore, 2004; Hippel, 2005; Sharpe, 2010; Dovey et al., 2014; Crossick and Kaszynska, 2016; REACT, 2016, p. 12–16; Gabriel et al., 2017). The health arena is one in which this broader scope of open activity is now finding expression, arising in response to failures in the dominant models of health innovation, concerning, for example, time-lags in the development of new products and services, the rising cost of innovation work, disconnect between innovation focus and identified areas of high priority, and inconsistencies in how, or which, innovations are taken up (Dixon-Woods et al., 2011; Gabriel et al., 2017). These points of failure are strongly linked to the fragmented and siloed nature of healthcare systems, introducing gaps in multi-stakeholder understanding of health needs and leading to the dominance of particular professional, disciplinary, or sector-led positions where a broader range of perspectives might be productively taken into account. In exploring the scope of open innovation in response to these failings, three key themes emerge for consideration: we can ask what it means to be “open” in terms of who participates in innovation, what those innovation activities might be, and the what the wider context of those innovation activities should be.

### 1.2. Perspectives on Open Innovation in Health

#### Participation in Open Activities

The health arena is well-suited to pushing the boundaries of participation in health innovation, with patients, carers, businesses, universities and research institutes, non-governmental organisations, community groups, and local or national governments amongst those identified as now critical in tackling the most resistant health challenges (e.g., Hippel, 2005; Bullinger et al., 2012; Gabriel et al., 2017). This is understood as key to diversifying the drivers and enablers of innovation, and, most critically, capturing the full plurality of valuing practices (cultural, social, economic, and so on) that are at play in concepts of health. Highly unsuitable to a generic approach, each open initiative faces the challenge of identifying who the right partners might be for identifying an innovation focus and delivering a response to identified challenges. In opening up innovation to a greater diversity of inputs, the demands being made of innovation work are also now changing. Greater participation is broadening more conventional technological and economically motivated interests to capture the demand for a greater variety of products and services, but also system reform, new training approaches, organisational development, policy change, and so on (Gabriel et al., 2017). With this greater recognition of the diverse drivers of innovation, the premium of the narrowly defined “solution” may also be seen to diminish; so, too, the value of novelty, where a repurposing and repair of existing products or services may bring greater benefits to a wider range of stakeholders. As such, these reappraisals are leading to an expansion in the types of participation as a function of the innovation process; initiatives are now found that introduce open principles into a variety of established innovation stages, including problem identification, R&D, and innovation adoption and diffusion (Gabriel et al., 2017).

#### Support for Open Activities

With its focus on working outside of more established sectoral or disciplinary practices, adopting an open orientation means rethinking the nature of innovation programming. Key factors that constrain open innovation in the health arena range from organisational challenges in introducing innovation activities, different legal, and regulatory environments that make cross-sector collaboration difficult, challenges associated with identifying, forming, and maintaining external partnerships, to the lack of venues or opportunities to engage knowledge diversity (e.g., Wass and Vimarlund, 2016). As such, open working can often be strongly limited because of the many different ways it will run against the grain of established sector practices. For example, whilst there is extensive scope for open innovation in medical technology, most open innovation in the industry is still limited to a “one way” exploitation of others' knowledge at the earliest stages of the innovation cycle (Wass and Vimarlund, 2016). The challenges faced in overcoming barriers to open engagement are such that many initiatives need to build a comprehensive innovation environment in parallel to the dominant innovation model for that sector. This might include, for example, developing (1) a stacked approach to project development, aligning multiple innovation stages together to support innovative work that will otherwise struggle to access conventional innovation support; (2) multi-stakeholder sector scoping to define critical themes and support the creation of lasting partnerships in advance of undertaking innovation activities; (3) bespoke brokerage and collaboration support to manage new cross-sector teams; (4) and a sensitivity to local and regional conditions in order to support wider innovation diffusion and adoption beyond established mechanisms (e.g., Robles et al., 2015; Malmberg and Vaittinen, 2017; Transform Ageing, 2018).

#### Context of Open Activities

Accompanying a greater diversity in participation and the development of new approaches for bringing extensive open innovation activity to fruition is a reappraisal of the wider focus and contexts of health innovation itself. Open methods are playing an important role in broadening that focus, opening conversations out-beyond traditional health priorities to raise issues around mobility and access to health services, education and health training, the social and cultural contexts of health, public health and longer-term health promotion, and so on. This broader conception of what is at stake in the health arena not only crosses conventional forms of innovation programming, but also raises the question of what open methods developed outside of the health arena might offer health related work in terms of new insights: if the strength of open approaches lies in managing interactions between non-traditional partners, then important perspectives on open innovation around health matters are likely to be found elsewhere as well. This is to recognise that an “open orientation” is not only highly generative, but hard to contain in that the continued questioning of perceived boundaries comes naturally to it. This is no better represented in how open initiatives are beginning to define new multi-sector conceptual spaces (such as the digital social innovation or smart cities paradigms) and give rise to collaborative methodologies with wide applicability across different fields of interest. Here, it is the opportunities both within and between fields of interest such as health, economics, energy, environment, democratic space, science, education, culture, employment, and so on, that are seen as a vibrant and productive space for innovation (Bria and Baeck, 2015; Robles et al., 2015, p. 122; Senior, 2018a).

## 2. Five Principles for Open Ecosystem Innovation

The conceptual and practical implications of opening up participation in innovation, developing new support mechanisms for open partnerships, and broadening the contexts in which open initiatives operate have been laid bare by the Knowledge Exchange Hubs for the Creative Economy funded by the UK's Arts and Humanities Research Council (AHRC). Operating between 2012 and 2016, the four AHRC Hubs demonstrated the many ways in which new cross-disciplinary and cross-sector partnerships could be built around a creativity agenda as applied to health and well-being, culture and heritage, publishing and documentary, open data and digital democracy, collaborative place-making, smart public services, gaming and entertainment, and much more besides. Together, they sought to enable broader participation in innovative research, policy, service, and product development (Creativeworks London, 2016; Design in Action, 2016; Dovey et al., 2016; REACT, 2016; The Creative Exchange, 2016; AHRC, 2017). Further developing this author's analysis of the AHRC Hubs programme (Senior, 2016, 2018a,b,c), five key principles for practicing what will be termed here “open ecosystem innovation” can be discerned from their work.

### Principle One: Working With Distributed Knowledge

The first principle is to recognise the highly distributed nature of knowledge production and mobilisation in the twenty-first century. With its broad creativity focus, the AHRC Hubs demonstrated the potential for a wide variety of cross-sector partnerships across the programme's nearly two-hundred projects. New partnerships were formed from amongst 73 different academic subject areas (across the arts, humanities, and sciences) and 47 different areas of creative economy work, drawing in participation from across the private, public, and voluntary sectors (Senior, 2018a). This work revealed the many ways in which dominant conceptions of professional boundaries can mask the extensive multi-disciplinary, cross-disciplinary, and non-disciplinary expertise that project partners can bring to project work, or the different ways in which participants understand their expertise to be constituted, mediated, and mobilised. Indeed, points of collaborative entry would often reflect interests that transcend formalised or non-formalised bodies of knowledge (not least those in narrative experience, place making, creative provocation, experience design, material culture, and so on). The notion of “crowding diversity” emerged from the programme as a model of drawing together the wide-ranging expertise needed for innovative project work, one in which a Hub's activities serve to enable that access and deliver greater parity within cross-sector partnerships (REACT, 2016, p. 17). With this focus on the strength of cross-sector collaboration as a generator of innovative new work that stands apart from the professional or institutional activities of project partners, a programme-wide consensus emerged on investing Intellectual property (IP) directly in project partnerships (whilst respecting partners' prior IP). This differs from dominant models of sector-led knowledge exploitation, such as in the UK university sector in which where IP emanating from the work of academic employees would be retained by their institutions (and often with negative consequences for research collaborations with external partners; Virani, 2015a).

### Principle Two: A Comprehensive Approach to Innovation

The second principle is to recognise that high levels of uncertainty associated with driving innovative new cross-sector partnerships requires action to limit value loss from the innovation process as a whole (e.g., through more effectively connecting different stages of the innovation cycle together), to reduce unnecessary risk for project teams across an innovation cycle (for example through supporting extensive peer-to-peer learning and streamlining administrative and contractual processes), and to help capitalise on the highly generative, and often unpredictable, nature of innovation work (e.g., by enabling partnerships to iterate project work and identify the right trajectory for its development) (Senior, 2018b). At the heart of this is a question about effective innovation programming. Whilst the AHRC Hubs differed strongly in their motivations for engaging in innovation work, a common framework emerged that exposes some of the fundamental needs of open innovation practice (Senior, 2018b, p. 12–16). The framework was originally proposed by the Design in Action Hub to describe their own innovation model (Woods M. et al., 2015), and is characterised by a five-stage innovation cycle (Scoping-Interpretation-Ideation-Formation-Evolution; the SIIFE model). Initial Scoping work defines Hub activities aimed at idea discovery and concept development; centred around the identification of critical challenges for target communities, sectors, and disciplines, this stage aims to establish the right targets for innovation work and potential project partners. An Interpretation stage that follows helps further develop and frame Scoping work into sector-relevant calls that anticipate productive areas of multi-sector collaboration. Only in stage three—Ideation—are participants first assembled around a themed call, with careful brokerage and curation of participants required to build a strong “ideas base” from which new partnerships might emerge. Competitive entry into Stage four—Formation—leads to a period of intensive project development, with an important role established for tailored project support, cohort-based peer-to-peer-learning, and expert-led R&D. Finally, an Evolution stage marks the transition from prototype development toward advanced project realisation or market launch, where the Hub helps teams to tap into external funding, and support mechanisms.

### Principle Three: Responding to Your Network of Partners

The third principle is to recognise that whilst a generic innovation framework might capture much of what is needed in adopting an open orientation, it is the development of a strategy tailored to the particular interests, needs, and pressures of a target innovation landscape that brings relevance to its application. Four different strategies emerged from the AHRC Hubs that this author has framed in terms of the SIIFE model (Senior, 2018b, p. 18–37): these included a Seeding Innovation strategy for the Creative Industries aimed at increasing and diversifying participation in innovation work through a large number of small-award, rapid development innovation vouchers (Creativeworks London, 2016); an Action Research strategy that centred project development on emerging academic research, allowing more experimental projects to emerge and trigger new projects over a longer time-frame (see also The Creative Exchange, 2016); a Design-led Business Development strategy focusing on the generation of new businesses through investing in a smaller number of projects and providing ongoing support through to market launch (Design in Action, 2016); and the practice of Cultural Ecology with its focus on a high-value and ambitious R&D programme embedded within a community of creative practitioners and academics, so strengthening a wider engagement around new ideas, innovative approaches, and project insight (REACT, 2016). In this way, the AHRC Hubs demonstrated the flexibility of a common underlying innovation framework. With each Hub targeting different innovation challenges, the programme as a whole resulted in a highly diverse body of work, including new products, services, research, and artistic outputs, job creation, teaching methods, and so on—all indicative of the highly generative potential of an open orientation (e.g., Senior, 2016, p. 27–29).

### Principle Four: The Importance of Mediation

The fourth principle is to recognise that the collaborative production at the heart of open innovation requires mediators with great sensitivity to different sectoral practices. Effective mediation can be critical in the brokerage and well-being of new partnerships, the successful development of new projects, and the effective navigation of institutional systems and processes that make such work possible (Senior, 2018b). Each AHRC Hub developed its own form of intermediary role, reflecting their particular innovation ambitions and the sectors and disciplines involved: first, the “cultural and creative knowledge broker” working to help different partners match interests, expectations, and compatibilities prior-to, and throughout the collaborative process (Virani, 2015a; Creativeworks London, 2016, p. 34–37; Senior, 2018b, p. 18–21); second, the designer introducing tailored design methods to support partners through a process of exploring, iterating, and testing new collaborative ideas (Design in Action, 2016, p. 6–8; Senior, 2018b, p. 22–27); third, the innovation-enabled PhD candidate, in which managing cross-sector work formed a central part of a PhD training programme and created a route for new research agendas to shape project development or open up new project trajectories (The Creative Exchange, 2016; p. 30–32; Senior, 2018b, p. 32–37); and, finally, the creative producer, that most fully brought together multi-sector and project management expertise into a single dedicated role (Dovey et al., 2014; REACT, 2016, p. 18–19; Senior, 2018b, p. 28–32). Here, creative producer activities include generating new connections between people and institutions, brokering collaborative opportunities, protecting new partnerships from damaging bureaucratic or administrative hurdles, supporting partnerships through the collaborative journey, and providing creative and practical advice^1^. The more encompassing open initiatives become, the greater value such dedicated roles may bring, a recognition that effective cross-sector working will often exceed a “skill set” that can be added to existing professional roles.

### Principle Five: Operating Sustainably

The fifth principle is to recognise that establishing new forms of open collaboration across sectors is a long-term culture-change project that requires an ongoing and sustainable presence. The “Hub” concept is important in this regard (Senior, 2018c). Less a bricks-and-mortar entity with a centralising effect on activity around it, a Hub can also be understood as an interface operating within a network of partners, delivering a programme of work that serves as a focal point for key strategic, research, and production activities at a given time. In this author's analysis, the AHRC Hubs revealed a number of core aims that underlie this way of working (Senior, 2018c). Most centrally is working to become embedded within multiple sectors whilst attaining the degree of autonomy needed to hold a critical and functional distance from them, i.e., establish a leadership role that can help forge a shared trajectory amongst different sector partners united by common underlying interests. Building on this, an effective Hub should strive to deliver an innovation programme with strong governance that can support the delivery of time-limited, targeted outcomes in response to identified innovation challenges—a systematic (rather than an *ad hoc*) response to a complex innovation landscape. Within this, a Hub must be able to develop the key brokerage, administrative, and support roles needed to build new forms of parity-driven collaborative partnership. Finally, an effective Hub should strive to become operationally agile, one able to embed learning arising from its own work and so develop a consistent and effective commitment to a target innovation space. This requires achieving sustainable operations that can break free from the limitations of time-constricted institutional or sector-led funding. In essence, this Hub model operates by pulling the essential components of co-production together, shifting the centre of gravity away from individual sectors. Put another way, the act of “being a Hub” is perhaps the biggest single challenge faced; it means establishing a Hub structure and culture, building effective partner networks, driving culture change within partner sectors, and striving for lasting influence beyond its immediate network.

## 3. A Model of Knowledge For Open Ecosystem Innovation

These five guiding principles point to a configuration of open activities that are maximally sensitive to (1) knowledge diversity in innovation work; (2) the consequences of adopting an open-orientation across all stages of innovation programming; (3) the value of deepening and broadening the targets of innovation activity; (4) the role of mediation in supporting cross-sector partnerships; and (5) the importance of operating in an adaptive and sustainable manner in the long-term. The claim here is that together they configure open innovation in a way that is radically decentralised from the interests of any given single organisation, discipline, or sector in the pursuit of innovation needs of shared interest—what I have termed open ecosystem innovation. Whilst still in its infancy in an innovation context, the “ecosystems thinking” referred to here tries to capture a specific instance of living organisms within a shared habitat and the patterns of value that coordinate those lives (e.g., Fuller, 2005; Sharpe, 2010; Markusen et al., 2011; Pratt, 2014; Senior et al., 2015; Crossick and Kaszynska, 2016). As such, it concerns more than just a question of *who* is involved, but the quality, dynamics, and time-evolution of their interactions together, as captured in the five principles. Looking at them again through a single lens—the status of knowledge—adds important nuance to this ecosystems perspective and helps reveal the wider ramifications of adopting such an account.

In the rise of the innovation economy, a conceptual model of knowledge has emerged that sees “knowledge work” as an extension of an economy of physical transactions, a refashioning of traditional manufacturing industries (Pratt, 2014). Imagined as a form of “physical good” (e.g., ideas as commodities, say in the form of intellectual property or patents), the “thingness” of knowledge renders it suitable for transfer or exchange^2^—the language of the innovation pipeline, of knowledge inflows, outflows, spill-overs, and pipeline couplings^3^. The conceptual ease of this “physical” model, however, has proven highly consequential for how knowledge is made subject to innovation policy. Following the arguments of Pratt, we see (1) an emphasis on knowledge outputs that can be readily assessed (tracked and compared) against key performance indicators; (2) the notion of creativity, as sited with[in] specific individuals or professions—the “you have it or you don't” model that serves as the basis for targeted (and, therefore, exclusionary) forms of funding and support; (3) infrastructural conceptions of innovation as self-contained activities that need merely to be implemented, ready subjects for streamlining and efficiency drives; (4) and the dominance of spatial targets for policy intervention, i.e., a focus on bricks and mortar co-working spaces, co-location, and regional clustering—forms of economic market place where knowledge can be traded as physical good. Ecosystems thinking suggests a very different account of knowledge. Rather than a thing to be transferred or exchanged, knowing is fundamentally relational, and, therefore, highly situated, and context-dependent (e.g., Pratt, 2014). In this way, an alternative model of knowledge can be proposed.

Looking again at the first principle for a model of open ecosystem innovation, it concerns not just sensitivity to knowledge diversity in innovation work, but also how knowledge is constituted within communities of practice (rather than “isolated” within individuals), so directing attention both to different forms of knowing (tacit, skill-based, formal, and informal; e.g., Facer and Enright, 2016), and the activities and values between individuals through which knowledge is mobilised and stabilised or destabilised. From this, an ecosystem perspective opens up, rather than closes down, the search for who should be engaged in innovation work and what it means to access “sources” of knowledge in the context of innovation programming. Looking again at the second principle, which concerned sensitivity to the wider contexts in which new innovation programming is planned, this particular lens focuses attention on how patterns of knowing (that help coordinate the life of an ecosystem) must be understood as pre- and post-dating any planned intervention (such as driving novel forms of collaborative partnership). This should orient open innovation programming to ask what activities might be needed prior-to and following innovation activities to enable that work to emerge from and integrate back into active patterns of knowing and knowledge work. The third principle concerned a sensitivity to the scope of innovation activities demanded of an open orientation. Critically, patterns of knowing—and the logics they entail—are always plural. Thus, innovation work might be embedded in an “economic logic” (currency as one *way* of coordinating action in an ecosystem), but it is not the only logic at hand; indeed it can be a limited driver for innovation when pursued at the exclusion of other, fundamentally entailed, logics. Put another way: ecosystems are not “for anything” (such as the economy), only the maintenance of the complex life that sustains it (Sharpe, 2010, p. 35; Pratt, 2014, p. 11). As such, a “healthy” open innovation ecosystem is one that enables different patterns of knowing (their values and logics) to be contested through innovation work, rather than compromised through a single dominating logic (such as economic growth).

The fourth principle concerned sensitivity to the need for dedicated mediation roles in the support of open innovation activities. An effective encounter between different ways of knowing (constituted through language, logics, values, power-relations etc…) can require more than just mere exposure to one another; it can require mediation. Rather than leaving potentially valuable interactions to chance, this points to the value of dedicated intermediaries who can meaningfully engage different sector cultures, and so help collaborative partnerships to inhabit (and ask questions of) each other's professional commitments and values. Critically, this is not a question of adopting a relativistic or anti-disciplinary stance on knowledge—it is precisely because different ways of knowing capture different surfaces of complexity that valuable insight can be gained through their contestation. Finally, the fifth principle concerned the development of an adaptive and ongoing ecosystem presence if innovation needs are to be met effectively and in the long-term. The relational nature of knowing renders it far-reaching, highly dynamic, and subject to tension between stabilising and destabilising forces. Achieving both a critical presence and duration (see also Pratt, 2014, p. 12) to understand and influence those knowledge networks, being highly reflexive of changing ecosystem states, understanding the potential ramifications of innovation activities throughout the full life of the ecosystem, *anticipating* future changes in the life of an ecosystem; all are required for this form of sustainable working. This reveals the difference between a “pipeline” innovation agenda that focuses primarily on generating “outputs” and an agenda that aims to create or sustain the wider conditions from which innovation activity can arise.

## 4. Dementia Connect: Scoping The Dementia And Creativity Arena

Following on from the AHRC Hubs programme, Dementia Connect asked how this approach to open ecosystem innovation might apply to an important cross-sector health challenge. With a view to exploring the AHRC's open ecosystem innovation principles, a highly contested multi-sector field of interest was chosen: dementia and the role of creative participation in delivering important health outcomes for those living with a dementia diagnosis. Core team members from the four AHRC Hubs participated in the project, serving key leadership, advisory, and research roles. A seventeen-member, cross-sector advisory board supported this core team in delivering all aspects of the project, which was conducted through the University of the West of England and the Foundation for Arts and Creative Technology (FACT, Liverpool). Underscoring the ethos of the principles in question, the approach adopted by Dementia Connect was to build a cross-sector network of partners spanning the “dementia and creativity arena,” consult them on the current state and possible future trajectories of the field in relation to open innovation activities, and to activate the network through funding new, innovative cross-sector partnerships. Together, these activities (detailed in section 4.1) served as a method to map critical actors, interactions, values, and aspirations (outlined in section 4.2) through which the five guiding principles for open ecosystem innovation activity could be assessed (see section 4.3); building on this work, a blueprint for open innovation ecosystem in this field can now be put forward (section 5).

### 4.1. Dementia Connect Activities

#### Dementia Connect Development Labs

Dementia Connect's centrepiece was a sequence of four full-day development labs, collectively addressing target themes linked to open ecosystem innovation through face-to-face interaction amongst the project's emerging partner network. Labs were used to identify current cross-sector challenges, opportunities, and best practice in the dementia and creativity arena; to develop new ideas for prototype products, services, and experimental research; to identify principles for increasing participation in creative activities and broadening access into innovation work; and to explore different sector-specific evaluative practices and impact-revealing activities. A lab consisted of a full-day programme of project development activities, typically for 20–25 participants and led by a creative producer. A Lab might draw on a range of different activities to probe a chosen theme and deliver on the Lab's aims, including brainstorming work, lightning talks and provocations, simple storyboarding, iterative project ideation, SWOT analysis (exploring the opportunities behind a project idea, its potential strengths and weaknesses, and the threats to its implementation), and Importance-performance analysis (IPA; helping to unpick key priorities and areas of over- and under-performance in a given field). Development labs were adapted to activate the project network in ways best suited to a theme's demands: a Lab might, for example, be built around a curated cohort of participants self-organising around shared interests (a form of “curatorial bricolage” (Virani, 2018); “leading lights” from the network invited to bring in their own team and project focus; or, an open-call beyond the network to bring in new ideas or test/challenge an emerging consensus. Insights from the Labs were also fed into research activities drawing on field literature and semi-structured informal interviews with sector partners to further contextualise or assess open ecosystem innovation insight emerging from the Dementia Connect project.

#### Dementia Connect Creative Voucher Scheme

Each development lab was associated with a competitive creative voucher (CV) scheme, a means of helping participants work together after a Lab event to prototype new ideas or conduct research as part of the Dementia Connect project. Totalling £20 K (£2–5 K per award), vouchers were essentially a means of nudging innovation around a given Lab theme. Historically, small voucher awards have proven a useful stimulus for projects that might not otherwise receive funding in traditional sector contexts or within the constraints of large award schemes (e.g., Virani, 2015a,b; Shiach et al., 2017): they can go far in supporting new work when attached to minimal application and reporting requirements (with time-saving and motivational consequences); they can unlock considerable in-kind support, a key sign of partner commitment that enables projects to go even further; and they are suitable for first-time entry into cross-sector collaborations whilst also helping more experienced, ambitious partnerships to work together in new ways—findings that were corroborated in our work for Dementia Connect (Virani, 2018). In the two Labs dedicated to the development of new project teams, two thirds of participants submitted one or more applications for a CV. More details on the CV projects developed through Dementia Connect can be found on the project homepage (Dementia Connect, 2018).

#### Dementia Connect Network

At the heart of Dementia Connect was a network of experts and advisors from a variety of different sectors. The four development labs and seven innovation projects funded drew on the expertise of 105 participants (including the project's core team and advisory board), including: 28 academics from 18 universities, partners from three innovation agencies, 22 arts, culture, and design practitioners, 3 national social care charities operating at least one care home, 7 participants from the National Health Service (NHS) and public health, 9 micro creative businesses, 19 charities working to improve care delivery, and 7 people living with a dementia and their care partners. The network was centred around Merseyside, with 66% of participants coming from the North West); a further 10% came from each of Bristol and London (reflecting the research base of the Dementia Connect team), with the remainder coming from across the UK (North East, Yorkshire / Humberside, West Midlands, South East, Wales, Scotland, and Northern Ireland). Development lab participants also included key partners from nationally operating networks, including the LAHF (London Arts in Health Forum), the AHSN (UK's Academic Health Science Network), the Dementia Engagement and Empowerment Project (DEEP), and National Museums Liverpool, amongst others. Critically, nearly 200 people living with dementia, their care partners, and front-line care staff (including participants from a number of different ethnic and cultural groups) were directly involved in the development, delivery, and evaluation of innovation projects. Dementia Connect activities were disseminated through its website and public showcases at FACT Liverpool, Liverpool John Moores University, Liverpool Life Science UTC (University Technical College) and the 2018 International Business Festival hosted in Liverpool.

### 4.2. Mapping the Dementia and Creativity Arena

Through the work of Dementia Connect, an outline of the dementia and creativity arena could be developed, addressing the scale of the dementia challenge, the changing dementia demographic in the UK, the role of arts and creativity as forms of dementia intervention, and the current state of the dementia innovation landscape.

#### The Dementia Challenge

There are currently 47 million people living with a dementia, a number expected to almost triple by 2050 to over 131 million (Prince et al., 2016; for the UK, see Prince et al., 2014); in most high-income countries, it is estimated that only 40–50% of people living with dementia have received a diagnosis (Prince et al., 2016, p. 6). Dementia is a descriptive term defining significant changes from a person's usual level of cognitive functioning, for example changes in recalling memories, finding words, recognising objects, carrying out practical tasks, or making considered judgements (Alzheimer's Society, 2017, p. 12–23). There are a number of underlying causes that affect the health of a person's brain in this way. Dementia can take many forms, with the most common being Alzheimer's-type dementia, vascular dementia, dementia with Lewy bodies, fronto-temporal dementias, and Parkinson's dementia. The disease course varies according to sub-type and a person's health status, but, in general, there is a slow progressive decline in functioning over a number of years through to the point where individuals are unable to survive without a very high level of personal support. With dementia onset, individuals also become vulnerable to the breakdown in their sense of self, which can lead to anxiety, confusion, low self-esteem, and often social disconnection and marginalization; the effects of dementia on friends and family can be devastating (Batsch and Mittelman, 2012; Kane and Cook, 2013). The underlying innovation drive—that dementia is likely to continue being an important focus of attention for the foreseeable future—is one that needs to be addressed: not only is there a need to respond to immediate challenges, but to anticipate possible future challenges (given that the configuration of dementia-related needs is going to change with each generation), and to put preventative healthcare measures in place where possible.

#### The Dementia Demographic

As diagnosis rates are driven up, and diagnoses made earlier (e.g., Mukadam et al., 2014), the dementia demographic increasingly includes those still leading active lives and living at home. Further, the number of people live with a diagnosis is closely matched by those taking on care responsibilities in the home, carers and families who often struggle to receive instruction or support, and shoulder two-thirds of care cost (Lakey et al., 2012; Prince et al., 2014). With longer periods receiving informal, extra, and domiciliary care, communities now face serious challenges in accommodating those living with a dementia diagnosis. This society-wide expression of the dementia challenge is reflected in the UK through the proliferation of organisations building critical support networks, sharing best-practice, generating collective insight beyond individual cases, and filling in gaps in social care. These have emerged to support people living with dementia (e.g., DEEP, the UK Network for Dementia Voices with over 68 groups led by people living with dementia), their carers (e.g., the National Dementia Carers Action Network or Together in Dementia Everyday, TiDE), friends and families (dementia friends movement operated by the Alzheimer's Society and now with the commitment of over 2.6 million Dementia Friends; Dementia Friends, 2017), for communities (e.g., the Dementia Friendly Communities movement with 196 signatories recognised by the UK Alzheimer's Society; Woodward et al., 2018), and for dementia-support organisations themselves (e.g., the Dementia Action Alliance, with over 7,000 member organisations across England; Dementia Action Alliance, 2019). Whilst sectors such as banking, retail, transport, and arts and leisure are working to build their provision to support a more dementia friendly world (including dementia awareness training for staff and adjustments to business processes or work programmes (e.g., Camic and Chatterjee, 2013; Wootten et al., 2016), the shift in sites of care from predominately care home environments to include family homes and communities is a challenge without historical precedence. It is a challenge that state-supported and market-driven health innovation is likely unable to address alone, with an important role envisaged for even the smallest social enterprises and community groups (McNeil and Hunter, 2014; All-Party Parliamentary Group on Arts Health and Wellbeing, 2017).

The foundation for recognising the personhood of someone living with a diagnosis, and, therefore, the patterns of behaviour that constitute that life, is one of fundamental human rights, as recognised by the World Health Organisation: “People with dementia should be empowered to live in the community and to receive care aligned with their wishes and preferences” (WHO, 2017, p. 22; also, Equality and Human Rights Commission, 2011). The affirmation of this status comes in light of the greater risk of breaches in human rights present for older people dependent on care services (Equality and Human Rights Commission, 2011, p. 19; Boaden, 2016), but also the ways in which increasingly timely diagnoses now allow people to consider the care they wish to receive and to which they have the right (placing a degree of emphasis on consumer empowerment and innovation). Underscoring this is a broadening of “personhood” as the lens through which dementia has been addressed by research and practice to include perspectives on citizenship, a position that opens up discussion on issues of discrimination and social inclusion, one that is more inclusive of the full complexity of living with a dementia diagnosis today (e.g., Bartlett and O'Connor, 2007; Kontos et al., 2017). Seen through the lens of Dementia Connect's work, this highlights the need to re-think the “patient status” of people living with dementia, the complexity of attaining ethical research approval when partnering with universities in collaborative work, the role of fair compensation and IP protection for all participants, and how informed—as well as continued—consent is to be managed as a function of the dementia journey.

#### Dementia Interventions

At the moment, there is no cure for dementia. Pharmacological interventions aim to improve cognitive functioning or to reduce distressing symptoms, but there is currently no treatment that can convincingly alter the course of the underlying condition (in relation to Alzheimer's for example: Anand et al., 2014; Khoury et al., 2017). With dementia progression, sustaining contact and communication (both verbal and non-verbal) becomes ever-more important in maintaining quality of life and well-being. Here it is significant that people's artistic, imaginative, and emotional capacities can remain strong for years after dementia onset. A growing body of evidence now reveals that arts-based and cultural interventions can elevate people above the stresses of dementia, slow degeneration, improve memory and communication, help drive social interaction and (re)-connection, and provide an important means of self-expression (e.g., Beard, 2011; Gould, 2013; Windle et al., 2014, 2018; Basting et al., 2016; Young et al., 2016; Dowlen et al., 2017; Windle, 2018). The advantage of non-pharmacological interventions is multiple, with few, if any, negative side-effects and a positive impact that can even exceed those of pharmacotherapy intervention (Herholz et al., 2013, p. 1236). Whilst strengthening the evidence base remains a key priority (Windle et al., 2016; Gray et al., 2017; Thomas et al., 2018), the wider arts and well-being agenda is now receiving more attention from both national and devolved governments (Department of Health Social Care, 2016; All-Party Parliamentary Group on Arts Health and Wellbeing, 2017; Arts Council of Wales, 2018).

If it is to respond to the needs and rights of those living with a diagnosis today—namely, remaining independent for as long as possible, and having choice and control over their lives through all stages of their dementia journey—a focus on dementia and creativity must expand its concerns beyond “creative activities” alone. As affirmed by Dementia Connect's development lab activities and the DEEP Participation creative voucher (DEEP, 2017a), such an agenda must capture a commitment to a person and citizen-centred vision of creative engagement, one that understands creativity as fundamental to well-being and social health but also places it in the context of human rights. Working with different forms of expertise, creativity, and value amongst people living with a diagnosis (and their care partners and communities) will mean simultaneously addressing the keys to meaningful participation: support, enablement, and accessibility. This means addressing challenges faced by those who are not active or well-supported; the need to ask how both traditional and emerging practices (e.g., digital practices) might help us rethink how, when, and where creative activities can take place; and a recognition that a creativity agenda can only succeed if our models of care, mobility, information provision, community support, and the designed environment are considered in interaction with it. Once again, this is to emphasise how an effective creativity agenda must stand with, rather than apart from, the realities of day-to-day living. That people living with a diagnosis must now play a more central role in the design of support services is now gaining recognition, with many examples of real-world application (Woods et al., 2013; East Dunbartonshire Council, 2014; Tsekleves et al., 2015; Woods L. et al., 2015; Zeilig et al., 2018).

#### Dementia Innovation Landscape

It is in this way that those living with a diagnosis, their care partners, medical researchers, front-line staff, community partners, artists, creative enterprises, and so on, all have a recognised—but different—part to play in delivering a higher quality of life for those living with a dementia. The exploration of new ways of working across these different perspectives, however, is still in an experimental phase, largely characterised by isolated collaborative projects, and with very few examples of coordinated or sustained (ongoing) programming in place^4^. Indeed, whilst many of the conditions for promoting dementia-related innovation are now present in the UK, and considered favourable in relation to other G7 countries (ADI GCA. Dementia Innovation Readiness Index, 2017), policy is still operating within largely conventional models of innovation, i.e., in relation to medical, technological, or social care innovation silos. The current “state of play” might be best understood as isolated pockets of activity that leave untouched key systemic barriers that prevent critical resources, key decision makers, stakeholder groups, and diverse sites of innovation being productively brought together in the long-term.

If a meaningful dementia and creativity agenda that can stand alongside the day-to-day realities of living with a dementia diagnosis is to be developed, then it is these tensions and potentials that need to be better understood. Working with different groups to understand both how they are responding to the dementia challenge and see themselves in relation to the dementia and creativity arena as a whole was a key activity of the Dementia Connect project. Engaging its wider network, a number of different group perspectives (“views from”) were developed, including those from the dementia experience, academic health research, arts and health practice, care home provision, clinical commissioning within NHS England, community organisations, and social-enterprises. In enabling these conversations, the Development lab and CV scheme could then support innovative collaborative projects to engage these different perspectives around a shared interest. This approach to surveying the field was critical not only in assessing the value of open ecosystem innovation principles, but also in gaining practical insight into what an innovation blueprint for the field might look like. One example of the learning developed through this approach (concerning health commissioning activities in England) should serve to illustrate the challenges faced in working across sectors in this arena and reveal some of the real-world potential for cross-sector innovation activities in the future.

#### Case Study: An Innovation Need

The “view from” described here concerns health commissioning activities within the NHS. In England, Clinical Commissioning Groups (CCGs; operating as part of NHS England) play a central role in commissioning local healthcare services (The King's Fund, 2017). Any service provider that meets NHS standards and costs (including social enterprises, charities, and private sector businesses) can receive a commission. Whilst the evidence base for the value of arts and cultural engagement in delivering health outcomes grows, it continues to be a very challenging commissioning environment for such work (Bagwell et al., 2014, p. 23). Increasingly, a number of health policies are being put in place that offer considerable potential for delivering on a dementia and creativity agenda, including health coaching, integrated personal commissioning, social prescribing, and personal health budgets. The move toward a social prescribing model is particularly interesting here, a recognition of the value non-clinical interventions bring to the delivery of health outcomes (Ward, 2016).

Whilst this might constitute the underpinnings of an active arts innovation agenda, the route to commissioning is fraught with challenges: a key challenge concerns visibility, with high-levels of arts and cultural sector fragmentation introducing barriers to effective communication and exchange. Many voluntary and arts organisations falling below the radar of commissioning are faced with the task (often beyond their resources) of re-organising, clustering, and lobbying in order to gain visibility. In corollary, commissioners can feel isolated in the task of finding promising new programmes or projects in their region; a second important challenge concerns procurement, with the persistence of approaches that are inappropriate to arts and cultural work, approaches that use highly contestable models of value and evidence. An “air of mystery” can surround the commissioning process as a result, with external organisations left questioning how agendas are set and decisions made; a third challenge concerns the sheer breadth of innovation activities—beyond the arts and across conventional silos—required if the promise of social prescribing is to be delivered. For example, whilst new digital technologies may aid in connecting health practitioners to service users through online social prescribing platforms, the valuable services they point to may be best supported through more traditional forms of artistic and practice-based engagement. Similarly, the development of disruptive new products and services might be the right goal to pursue in filling a commissioning gap, but so too a refinement or repurposing of what is already in place. All these challenges apply limits on effective commissioning, each further compounded by considerable regional differences in NHS structure (a consequence of the UK's devolution agenda) and the ongoing challenge of integrating health and social care practices nationally.

As such, commissioning may benefit from new cross-sector operating platforms that can help broker relationships with innovative, trustworthy partnerships, those developing new work with appropriate evaluation measures and pathways to wider adoption in place (e.g., Harris and Rowley, 2017, p. 12, 18). Through Dementia Connect's Development labs, a number of proposals emerged that speak to many of the issues around building capacity for social prescribing, proposals that were then refined, and prototyped through the CV scheme. Whilst only scratching the surface of the social prescribing challenge, they do reveal the potential for new ways of working and for developing a joined-up prescribing approach. Seen together, these three voucher projects reveal important connections that will need to be drawn between creative social care (e.g., The Activity Academy), post-diagnosis support (e.g., Drawing on Strengths), and information services (e.g., What's on for Dementia) if an effective approach to social prescribing is to be developed. They also reveal the value of supporting a wide variety of innovation-orientations and supporting diverse cross-sector partnerships.

The first creative voucher project—Activity Academy—was a partnership between Widnes Super League Rugby club, Halton Clinical Commissioning Group, and the national social care charity “Community Integrated Care.” The team asked how community-based creative resources might bring added value to person-centred care home routines where, traditionally, creative engagement with residents is considered a time-restricted activity delivered only by dedicated activity coordinators. In the Activity Academy, the team brought together leading regional figures in creative engagement and social care best practice to deliver an event for more than fifty frontline care home staff and managers in the Liverpool City Region. “Espresso training” activities and short practical workshops (Inspiration Stations) guided participants through the delivery of chair-based exercises that promote mobility in older people and the use of poetry, literature, and music to engage with residents and support reminiscence work. Post-event questionnaires pointed to an increased understanding of, and commitment to, person-centred support amongst participants, with care home managers reporting new activities as part of their services 4 months on. Activity Academy was a finalist in the 2018 UK National Dementia Care Awards.

A second creative voucher project—Drawing on Strengths—focused on the earliest stages of the dementia journey, the immediately post-diagnosis period. They recognised that whilst participation in creative activities can be an important route to making sense of changed circumstances, re-affirming personhood, and opening routes to sharing time with loved ones, too few clinicians direct people toward creative activities as part of the social prescribing agenda. Further, a dementia diagnosis centres on identifying reduced memory performance and the loss of cognitive abilities, a deficit-focus that can mask the rich interests, capabilities, and creative ambitions that can form the basis of meaningful creative participation. In response, the Drawing on Strengths project team—bringing together an NHS psychologist, a multi-disciplinary artist, an Arts and Health network, and an academic researcher of arts and devolution—developed a paper-based tool that can help someone with a dementia diagnosis build a snapshot of the creative, social, and community assets in their lives and build it into their care journey as part of the NHS Mersey Care's existing offer of post-diagnostic support. The tool was co-designed with 62 people, including those who have received a dementia diagnosis, their care partners, and dementia advocates.

An overarching challenge, namely limited public information on the availability (and suitability) of local creative activities for those living with a diagnosis, was addressed by a third creative voucher project—What's on for Dementia. Here, the team asked how people living with dementia might be helped to identify appropriate creative activities in their local area, whether through self-identification or as part of social prescribing. Through a partnership between the Uses of Arts Lab at Liverpool John Moores University, Welcome2Liverpool (a micro-enterprise), BBC Radio Merseyside, and NHS Liverpool Clinical Commissioning Group, a “what's on for dementia wellbeing” service was prototyped off the back of an existing, free phone app offering a real-time guide to events across the Liverpool City Region. Building on research that mapped community resources offering dementia friendly well-being activities, workshops were run with arts organisations, clinicians, app developers, and people living with a dementia to develop accessible design features for the service.

### 4.3. Assessing the Five Principles of Open Ecosystem Innovation

With this broad outline of the dementia and creativity arena in mind, the applicability of the five open ecosystem innovation principles developed through the AHRC Hubs programme (see section 2) can be gauged. The first principle addressed the need to be open to knowledge diversity in innovation work. Living well with a dementia diagnosis today implicates a wide range of experiential, disciplinary and sectoral activities, in part a consequence of changing dementia demographics and the emergence in recent years of a strong dementia rights, person-hood, and citizenship perspective. The growing role of support networks across different assemblages of those affected by dementia brings an added dimension to the relational and situated nature of knowing. Whilst the value of crowding diversity holds true, there is still need to develop new forms of participation for people living with a dementia if they are to meaningfully shape an innovation agenda. Participation might include contributing to the scoping of innovation themes, participating in user-testing environments, but there also needs to be new opportunities to actively participate in (or lead) project development. Whilst progress is being made in co-design and co-creative approaches with people living with a diagnosis (Tsekleves et al., 2015; Zeilig et al., 2018), there is still a need to address the status of intellectual property and fair compensation for people living with dementia in innovation work.

In developing an innovation programme that can respond to the whole life of this dementia and creativity ecosystem (principle two), and with relatively little innovation support already in place, there is a need for no-less than a full-cycle innovation approach. This would include working to identify critical innovation themes, broker new partnerships, enable R&D, and build routes that support innovation adoption and diffusion. Here, a variant of the five stage Scoping-Interpretation-Ideation-Formation-Evolution (SIIFE) framework developed within the AHRC Hubs programme could suit this task, one with the following specifications and adaptations made: first, the introduction of a pre-framework support stage for multi-stakeholder-enabled community organisations that are already active in the field and constitute the foundation from which innovative new work might arise and be adopted in the long-term (an Ecosystem Investment stage); second, the specification of an extensive Scoping and Interpretation stage in response to the high levels of ecosystem fragmentation; third, the specification of a prolonged Ideation stage to help initial experimentation with new ideas in a field where an innovation orientation (and cross-sector working) is still an emerging practice; fourth, the specification of a substantive investment in R&D and collaborative support during the Formation stage, appropriate to the scale of the innovation challenge faced and the involvement of people living with dementia throughout the collaborative process; and finally, a full commitment to integrating advanced project teams into other forms of project guidance and funding support through the final Evolution stage, a recognition of this highly underdeveloped component of the ecosystem.

Turning to the question of a focus for this innovation programme, and asking how it might best respond to the different patterns of activity and value that coordinate the life of an ecosystem (principle three), it becomes clear that the goal of helping improve the quality of life for people living with dementia and those around them cannot be achieved through a single innovation-orientation alone. A mixed approach is needed if the plurality of ambitions for the field are to be delivered—a meaningful response to the complexity of living with a dementia diagnosis today. A further specification of the SIIFE framework proposed here would be the support for “mixed” cohorts of projects, with the Formation stage tackling issues common to operating in a new dementia-focused, cross-sector innovation environment, and the Evolution stage offering tailored support toward specific markets or groups of ends users (i.e., in private, public, or third sector contexts). There are likely important benefits to be gained from aligning, rather than segregating different types of innovation activity, helping bodies of cross-sector knowledge to be developed and innovation programming to be conducted across sectors. A final specification would be to build multiple points of entry and exit into the innovation programme's five stages, transforming the programme from a singular “pipeline” to one that can accommodate already existing projects at different phases of development, each able to enter the programme stage best suited to them.

The fourth principle was to recognise the role mediation can play in engaging these myriad different patterns of life that constitute an ecosystem. As an emerging focus for cross-sector collaboration, many, often fundamental, differences in sectoral practices are now having to be contested: these include different positions on the values and priorities of care, the role of the arts and creativity in health intervention, the enabling or disempowering status of new technologies, and the place of dementia within models of ageing, disability, and human rights. The fragmented nature of work in the field can mean that whilst there is often a will to understand different points of view, there is little in place to support such perspective taking. In this, we identify a need for cross-sector enabled mediators to play a variety of roles across the proposed innovation programme, including helping to broker partnerships, reducing administrative burden in collaboration work, supporting R&D activities, working to instil parity in collaborations, and helping new project teams to embody the sectoral challenges of their project partners, and so on. Whilst these forms of support for collaborative production might not yet be formally codified, it may be that pressure to develop such roles in the health arena is beginning to emerge.

Finally, the principle of needing to develop an adaptive and sustainable ecosystem presence if effective and lasting interventions are to be made. A project-based culture in the dementia and creativity arena currently dominates, one that can, at best, only respond to present challenges, and has little capacity to support future planning or to anticipate the impact of changing demographics and advances in medicine and technology, for example, on an innovation landscape. What emerges is the potential for a cross-sector operating Hub-led approach to develop leadership in the field and enable the transition from a culture of “making do” to one that can both comprehensively respond to immediate needs and anticipate future challenges and opportunities—a simultaneous “three horizons” model (International Futures Forum, 2019). This describes a small adaptive Hub model operating as an innovation vehicle that can (1) become enabled in, and operate across, multiple sectors; (2) build and maintain trusted partner networks; (3) deliver innovation programming that instils core values in the innovation work it supports (such as around human rights and person-centred care); (4) adapt to changing innovation landscapes as a function of its own work and the work of others; (5) drive culture-change in sectoral practices that obstruct cross-sector working; and (6) strive to operate beyond time-limited sectoral or institutional programming to deliver ongoing commitment to this important challenge area.

## 5. An Open Ecosystem Innovation Blueprint For The Dementia And Creativity Arena

**Figure 1:**
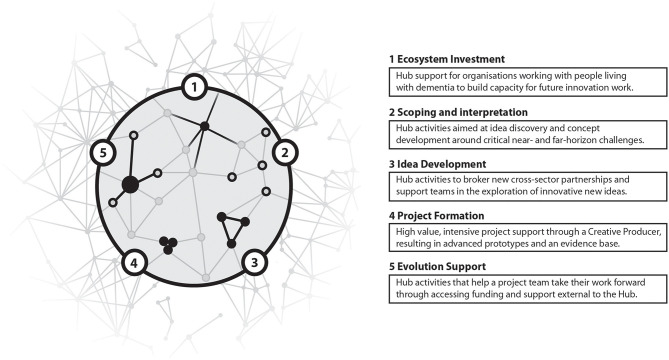
The blueprint for open ecosystem innovation developed through Dementia Connect for the dementia and creativity arena (detailed in section 5). Outlining a single innovation cycle, the blueprint describing five stages: (1) ecosystem investment; (2) scoping and interpretation; (3) idea development; (4) project formation; and (5) evolution support.

With this analysis in mind, a blueprint for an innovation programme in the dementia and creativity arena can be put forward, one that fully delivers on the open ecosystem innovation principles proposed. Whilst concrete recommendations for a specific innovation programme itself will be made here (along the lines of the SIIFE framework), the question of its delivery mechanism—i.e., the form of the Hub itself—is more open, and will be addressed in the Discussion (section 6). The blueprint is summarised in Figure 1.

### Stage One—Ecosystem Investment

The first stage of the proposed innovation programme is Ecosystem Investment, which has the aim of strengthening those partners within a regional ecosystem already serving key connecting, advocacy, mediation, and training activities. Funding would help such organisations to develop further capacity in the field and lay the foundations for new cross-sector partnerships that can deliver innovation activities to the communities who need them in the long-term. It is a key expression of shifting an innovation focus away from the delivery of targeted solutions toward growing an ecosystem that is itself “innovation ready”. Such a programme might be delivered through a rolling, competitive scheme for short-duration projects (of up to 4 months) each worth between £5–7K^5^. Funding might serve, for example, to help existing partnerships (already poised to advance their work further) to share best practice and key learning; run micro-residences to enable information gathering and engagement with key influencers; drive network intensification to promote organisational stability and resilience; develop evidence-based evaluation and dissemination activities; and trial delivery of existing activities at an increased scale of operation. This type of engagement would benefit the Hub through generating invaluable on-the-ground sector insight, enabling the identification of “leading lights” for future collaborative activities, helping to build user-testing environments for future innovation work, and strengthening the Hub network overall. Delivered at scale, such a scheme might have a transformative effect on the field.

### Stage Two—Scoping and Interpretation

This second stage marks the decision by a Hub to engage their partner network around a target theme, with sector scoping activities critical in establishing up-to-date, “live” multi-sector snapshots of pressing near- and far-horizon challenges or opportunities. The approach developed by the Design in Action Hub offers one suitable approach (Coulson and Woods, 2016; Design in Action, 2016, p. 6–8; Senior, 2018b, p. 22–27), with scoping activities placed prior to each innovation cycle through stakeholder workshops, design-led activities, formal/informal interviews, and multi-sector literature reviews. The Interpretation phase that follows Scoping work also requires a high degree of research excellence and sectoral knowledge from a Hub's core team to bring together insight from a complex, multi-sector landscape and shape it into sector-appropriate themed calls. Insight into broad themed areas suitable for new cross-sector collaborative partnerships has emerged through Dementia Connect's Development labs and research activities^6^. Identified themes included: stepping into a creative future (enriching creative, social, and inter-generational assets); building creative and dementia-friendly Environments; coping with transition (through creative, sense-making activities); empowering care (in the design and implementation of creative activities); innovation in information services (to advance new forms of information access, data ownership, and identity protection); building networks and communities; and experimental horizons (exploring productive alignments between contemporary dementia research and creative practice). Each theme could serve as a vehicle for implementing or advancing learning from areas of policy, clinical practice, and university research.

### Stage Three—Idea Development

Delivering on a themed call, the Idea Development stage would support the formation of new cross-sector partnerships prior to competitive entry into the Project Formation stage. For the AHRC Hubs, conducting Ideation activities over 1 or 2 days was deemed sufficient to form tentative new project teams, with post-event Hub support proving critical to refine project outlines and team composition (Senior, 2018b). In a dementia context, where cross-sector partnerships are more uncommon and partners may strongly differ in their experience of working with dementia, an initial period of collaborative engagement could be formative in helping new teams take some of the risks associated with project work in a difficult innovation space, test the strength of partnerships, and assess possibilities for longer-term collaborative activities. Hub support to broker teams and manage interactions will likely be valuable. The Idea Development stage could be delivered along the lines of Dementia Connect's own Development lab and CV model. Led by a creative producer, around 20–25 invitees would participate in an event designed to unpack the target theme, identify points of common interest, and develop new project ideas (outlining possible partners and roles, project incentives, and critical resources). A simple post-lab application (also open to external applicants) would enter teams into a competitive CV scheme for awards of £5–7 K (with a number of awards made for a variety of proposed project outputs). Project lead-time and delivery (around 5 months) would flexibly accommodate time-constraints faced by many in the field (particularly in the public and charitable sectors). Low-level project reporting combined with a collaborative project review would be mandatory at the conclusion of the Idea Development stage. Although an early developmental stage, project teams would be awarded IP, giving them confidence to take their ideas forward as a partnership (Senior, 2018c).

### Stage Four—Project Formation

Tied to the same strategic theme as the Idea Development activities, Stage four Project Formation would deliver project teams through an extensive programme of collaborative R&D, enabling teams to establish a quality benchmark for cross-sector working in the field. A competitive entry process would determine stage participation, one open both to project teams emerging from earlier stages in the innovation programme and those external to the Hub from the wider ecosystem. Open to different team ambitions, project outputs from this stage might include product prototypes, an innovative service redesign, experimental research work—projects that may respond to local-needs or demonstrate potential for scalability. At this stage in the innovation cycle, it would be the needs common to all projects in navigating a complex multi-sector innovation space that would be the key focus of a Hub's support activities. Here, the AHRC REACT Hub Sandbox could serve as an effective model for this stage of project development: a 3 month programme led by a creative producer built around a backbone of workshops, business development support, prototype iteration, user testing, industry consultation, and public showcasing events (REACT, 2016, 18–19; Senior, 2018b). A cohort-based approach—enabling stronger exchange between project teams, advisors, mentors, industry experts, project users, and so on—has proven valuable in this space (Senior, 2018b). Coming out of Stage four, project teams should be equipped to take their ideas toward advanced development and be better aligned to other investment and support opportunities external to the Hub. This model of support should be understood as bringing an additional “ecosystem perspective” to the provision typical of incubator programmes, such as through intensive, and more extensive, peer-to-peer exchange (Moreton and Dovey, 2013; Dovey et al., 2014). Stage four would aim to deliver 40–60 K into each new project with the commitment to the wider inclusion of people living with dementia throughout project development fully costed (covering travel expenses and with participation remunerated, and so on). A cohort of around five new partnerships (consisting of small teams of 3–4) would enable the intensity, but also the intimacy, required of a Sandbox process. Finally, and reflecting the same principles discussed for Stage three Idea Development, project IP would be held by project teams.

### Stage Five—Evolution Support

This final stage would see the Hub support teams toward advanced project realisation (such as market launch, or public-sector commissioning). Project teams entering into Stage five would be expected to have not only a viable project idea, but also the partnerships, assets, and evidence-base needed to convince potential funders of that viability. Projects from Stage four would compete for entry alongside those from the wider ecosystem at an equivalent stage of development.

Whilst a Hub might provide further financial support for project teams at this stage (particularly important for highly innovative cross-sector “benchmark” projects), a core activity must be to help engage project teams with other investment and support opportunities external to the Hub. For most projects, connecting into well-established forms of university R&D funding, business acceleration, tailored marketing, legal, and business advice for investment readiness, and so on, will be critical for their future development (including scaling, adoption, and diffusion activities). It is here in the wider ecosystem—beyond the Hub—where teams are better placed to access these forms of support (Senior, 2018b). Evidence from the AHRC Hubs programme indicates that continued and tailored Hub involvement through this transition (the well-documented “valley of death” between project prototype and “final product”) will be critical (e.g., Senior, 2018c, p. 25). Here, a Hub can help broker connections with additional sector partners and support programmes, help publicise new work through showcasing opportunities, facilitate project dissemination through sector-specific publications, and even help manage the reformulation of partnerships that might be required to take projects forward (Senior, 2018b, p. 30). Transitioning out of Hub support is very much project-dependent, a question of helping teams transition at the right time and pace for their ongoing development. Through the staged Hub support proposed, new project teams should be at an advantage in making that transition successfully. Further, with IP ownership in the hands of project teams, they will have greater control in how they decide to develop their work beyond the Hub. It is here, in Stage five, where new funding streams for the Hub itself might be developed, such as through taking an equity share in projects (see for example Design in Action, 2016, p. 40; Senior, 2018c).

## 6. Discussion

This paper has addressed the development of five guiding principles for open ecosystem innovation and their application to the dementia and creativity arena through the research project Dementia Connect. These guiding principles point to a configuration of open activities that are maximally sensitive to (1) knowledge diversity in innovation work; (2) the consequences of adopting an open-orientation across all stages of innovation programming; (3) the value of deepening and broadening the targets of innovation activity; (4) the role of mediation in supporting cross-sector partnerships; and, (5) the importance of operating in an adaptive and sustainable manner in the long-term. In exploring the application of these ideas to the dementia and creativity field, Dementia Connect undertook a wide-reaching scoping exercise grounded in an engagement with individuals, organisations, and communities active in this arena. As such, it's overall result—the Hub-led open ecosystem innovation blueprint described in section 5—is tied to insight from knowledgeable partners with an interest in seeing a more collaborative and meaningful approach to cross-sector work. This is a blueprint that now needs to be tested in practice.

In thinking to this real-world application, we can ask what an effective vehicle for this Hub-led innovation programme might be, one that respects the ecosystem thinking that underlies it. The cross-sector innovation leadership role proposed for the Hub is dependent on it becoming fully ecosystem-embedded whilst attaining the degree of autonomy needed to hold a critical and functional distance from individuals sectors, i.e., establish a position that can help forge a shared trajectory amongst different sector partners united by common underlying interests (see principle 5, section 2). One approach would be to align it with an existing programme of community-embedded dementia support that carries with it a similar ethos around open innovation. The Meeting Centres Support programme (MCSP) developed in the Netherlands in the 1990s, and now emerging in the UK, offers one such alignment (Brooker et al., 2017). The MCSP enables communities (typically of around 5,000 inhabitants over the age of 65) to design and operate a centre for the support of people living with dementia and their families. Open to contributions from all interested parties, centres are configured to the needs and opportunities of their locality, operating out of existing community sites. They serve as a social club, offer evidence-based post-diagnostic psychosocial interventions (physical, social, creative, and cognitive activities), “Understanding Dementia” meetings, and make regular opportunities available for people living dementia and their care partners to meet up with staff and talk through the changes happening to their lives. The programme now supports 144 centres in the Netherlands, with national infrastructure in place for local groups to bring new centres on stream, develop a business plan, and secure staff positions (Brooker et al., 2017, p. 8). Two UK centres are now in operation, one in Droitwich spa (Worcestershire) and Leominster (Herefordshire) (Brooker et al., 2017).

Once in place, Meeting Centres act to strengthen local networks of dementia-aware partners across a variety of different sectors, from the creative arts to retail. What the MCSP establishes is the community foundation on which an innovation Hub might be built—indeed, the foundation without which a Hub simply could not operate. With the demand for its services established, a Meeting Centre might then support an innovation Hub as a second developmental phase to its work. For example, run as a community interest company (an autonomous enterprise with broad fund-raising powers that enshrines a social mission consistent with the open innovation ethos proposed here), a Hub entity could work to open up access to different sector funding streams or lay the foundation for more bespoke forms of support. Targeting (and aggregating) external funding sources may enable key elements of the innovation cycle to be delivered (Stages 3-to-5 of the cycle, for example, find parallel in many current models of research and innovation support). An equity share in projects or the development of Hub spin-outs (e.g., focusing on innovation brokerage and consultancy) may generate additional income streams in the long-term, one route to a sustainable future. Returning to the example of health commissioning in England given as a case study in section 4.2, such a Hub could deliver the horizon-scanning activities that commissioners are currently hard-pressed to undertake; deliver the operational activities that link on-the-ground dementia realities with targeted innovation activity best suited to commissioning interests; and broker contact with innovative new partners. In corollary, project teams would be supported in developing and testing innovative new work, so building the evidence base needed to boost their visibility/credibility in the commissioning process.

Working with a variety of partners in the ecosystem, the establishment of a small, adaptive innovation Hub would signal a shift from delivering innovation activities that focus on “managing the present” to those that can also anticipate challenges and help build the communities of the future. The activities of the Hub would serve to further strengthen and expand the regional network of partners, potentially operating across multiple Meeting Centres in a region or a city to deliver impact more fully. This could be one route to embedding a Hub's work within its ecosystem and developing a sustainable innovation approach in the long-term, one that could keep responding to the complex reality—now and in the future—of living with a dementia diagnosis.

## Ethics Statement

This study was carried out in accordance with the recommendations of UWE's principles on Human Participant Research and the UWE Research Ethics Committee with written informed consent from all subjects. All subjects gave written informed consent in accordance with the Declaration of Helsinki. The protocol was approved by the UWE Research Ethics Committee.

## Author Contributions

TS led on the design and analysis of the Dementia Connect project, writing, and submitting the manuscript.

### Conflict of Interest Statement

The author declares that the research was conducted in the absence of any commercial or financial relationships that could be construed as a potential conflict of interest.

## Footnotes

^1^The role has since been codified as a professional masters programme at the University of the West of England (UWE Bristol, 2018).

^2^In the UK University sector, models of Knowledge Transfer continue to dominate thinking in interactions with external partners such as Industry (Dowling, 2015). The emergence of the Knowledge Exchange concept—an attempt to recognise forms of reciprocity in such partnerships—does little to tackle the underlying epistemic misconception of knowledge as “physical good.”

^3^The open innovation literature is itself commonly grounded in this style of thinking, invoking resource-based models of knowledge flow operating across well-defined internal and external organisational boundaries (see West et al., 2014).

^4^The Innovate Dementia Living Lab (with eight partners across North West Europe) is a rare example of such programming (Innovate Dementia, 2019).

^5^An example of this approach can be found in the Dementia Engagement and Empowerment Project's Influencing and Working Together grant schemes (DEEP, 2017b).

^6^The innovation briefs of the “Transform ageing programme” has proven useful in validating and framing this work (Transform Ageing, 2018, p. 8, 10–23).

## References

[B1] ADI and GCA. Dementia Innovation Readiness Index (2017). Alzheimer's Disease International and Global Coalition on Ageing. Available online at: https://globalcoalitiononaging.com/wp-content/uploads/2018/05/gcoa-adi-dementia-index.pdf (Accessed August 28, 2018).

[B2] AHRC, (2017). Creative Exchanges: The AHRC Knowledge Exchange Hubs For The Creative Economy Report. Swindon: AHRC. Available online at: https://ahrc.ukri.org/documents/project-reports-and-reviews/creative-exchanges-ke-hubs/ (Accessed August 28, 2018).

[B3] All-Party Parliamentary Group on Arts Health and Wellbeing (2017). Inquiry Report: Creative Health: The Arts for Health and Wellbeing. London: APPG. Available online at: https://www.artshealthandwellbeing.org.uk/appg-inquiry/ (Accessed August 28, 2018).

[B4] Alzheimer's Society (2017). The Dementia Guide: Living Well After Diagnosis. London: Alzheimer's Society. Available online at: https://www.alzheimers.org.uk/sites/default/files/2018-07/AS_NEW_The%20dementia%20guide_update%203_WEB.pdf (Accessed August 28, 2018).

[B5] Amin A. Cohendet P. (2004). Architectures of Knowledge: Firms, Capabilities, and Communities. Oxford: Oxford University Press.

[B6] Anand R. Gill K. D. Mahdi A. A. (2014). Therapeutics of Alzheimer's disease: past, present and future. Neuropharmacology, 76, 27–50. 10.1016/j.neuropharm.2013.07.004 23891641

[B7] Arts Council of Wales (2018). Arts and Health in Wales: A Mapping Study of Current Activity. Vol. 1: Analysis, Findings and Proposals. Cardiff: Arts Council of Wales. Available online at: http://www.arts.wales/arts-in-wales/celfyddydau-ac-iechyd?diablo.lang=eng (Accessed August 28, 2018).

[B8] Bagwell S. Bull D. Joy I. Svistak M. (2014). Opportunities for Alignment: Arts and Cultural Organisations and Public Sector Commissioning. London: New Philanthropy Capital. Available online at: https://www.ncvo.org.uk/images/documents/practical_support/public_services/cultural-commissioning/full-report-opp-for-alignment-arts-cultural-orgs-public-sector.pdf (Accessed August 28, 2018).

[B9] Bartlett R. O'Connor D. (2007). From personhood to citizenship: broadening the lens for dementia practice and research. J. Aging Studies 21, 107–118. 10.1016/j.jaging.2006.09.002

[B10] Basting A. Towey M. Rose E. (2016). The Penelope Project: An Arts-Based Odyssey to Change Elder Care. Iowa City, IA: University of Iowa Press.

[B11] Batsch N. L. Mittelman M. S. (2012). World Alzheimer Report 2012: Overcoming the Stigma of Dementia. London: Alzheimer's Disease International. Available online at: https://www.alz.co.uk/research/WorldAlzheimerReport2012.pdf (Accessed August 28, 2018).

[B12] Beard R. L. (2011). Art therapies and dementia care: a systematic review. Dementia 11, 1–24. 10.1177/1471301211421090

[B13] Boaden A. (2016). Fix Dementia Care Hospitals. London: Alzheimer's Society. Available online at: https://www.alzheimers.org.uk/sites/default/files/migrate/downloads/fix_dementia_care_-_hospitals.pdf (Accessed August 28, 2018)

[B14] Bria F. Baeck P. (2015). Growing a Digital Social Innovation Ecosystem for Europe. London: Nesta. Available online at: https://media.nesta.org.uk/documents/dsireport.pdf (Accessed August 28, 2018).

[B15] Brooker D. Evans S. Evans S. Watts M. Dröes R.-M. (2017). Meeting Centres Support Programme UK: Overview, Evidence and Getting Started. Worcester:Association for Dementia Studies. Available online at: https://www.worcester.ac.uk/documents/Meeting_Centres_Support_Progamme_Overview_evidence_and_getting_started_Conference_booklet.pdf (Accessed August 28, 2018).

[B16] Bullinger A. C. Rass M. Adamczyk S. Moeslein K. M. Sohn S. (2012). Open innovation in health care: analysis of an open health platform. Health Policy. 105, 165–175. 10.1016/j.healthpol.2012.02.009 22440194

[B17] Camic P. M. Chatterjee H. J. (2013). Museums and art galleries as partners for public health interventions. Perspect. Public Health 133, 66–71. 10.1177/1757913912468523 23308010

[B18] Chesbrough H. W. (2003). Open Innovation: the New Imperative for Creating and Profiting from Technology. Boston, MA: Harvard Business Press.

[B19] Chesbrough H. W. Vanhaverbeke W. West J. (2006). Open Innovation: Researching a New Paradigm. New York, NY: Oxford University press.

[B20] Coulson S. Woods M. (2016). Scoping: exploring a collective R&D process for entrepreneurs, microenterprises, and SMEs, in The 20th DMI: Academic Design Management Conference Proceedings. (Boston, MA), 435–459.

[B21] Creativeworks London (2016). Creativeworks London Evaluation Report: A Knowledge Exchange Hub for the Creative Economy. eds Matheson-Pollock B. I Jubb E. Morretto M. Riedel J. Roberts E. Shiach M.. Available at http://www.creativeworkslondon.org.uk/cw-news/creativeworks-london-evaluation-report-a-knowledge-exchange-hub-for-the-creative-economy/

[B22] Crossick G. Kaszynska P. (2016). Understanding the Value of Arts and Culture The AHRC Cultural Value Project. Swindon: AHRC. Available online at: https://ahrc.ukri.org/research/fundedthemesandprogrammes/culturalvalueproject/ (Accessed August 28, 2018).

[B23] DEEP (2017a). Guidelines for Meaningful Participation of People With Lived Experience of Dementia. Available online at: https://dementiaconnect.dcrc.org.uk/wp-content/uploads/sites/9/2018/08/DEEP-FINAL.pdf (Accessed January 30, 2019).

[B24] DEEP (2017b). DEEP Grants 2017 – England, Wales and Northern Ireland. Available online at: http://dementiavoices.org.uk/2017/05/deep-grants-2017-england-wales-and-northern-ireland/ (Accessed August 28, 2018).

[B25] Dementia Action Alliance (2019). DAA: Dementia Action Alliance. Available online at: https://www.dementiaaction.org.uk/ (Accessed February 12, 2019).

[B26] Dementia Connect (2018). Dementia Connect: Working Together to Build a Dementia Innovation Cluster. Available online at: https://dementiaconnect.dcrc.org.uk/ (Accessed January 30, 2019).

[B27] Dementia Friends (2017). Dementia Friends: An Alzheimer's Society Initiative. Available online at: https://www.dementiafriends.org.uk/ (Accessed August 28, 2018).

[B28] Department of Health and Social Care (2016). Prime Minister's Challenge on Dementia 2020 Implementation Plan. London: Department of Health. Available online at: https://www.gov.uk/government/publications/challenge-on-dementia-2020-implementation-plan (Accessed August 28, 2018).

[B29] Design in Action (2016). Design in Action: A New Economy of Knowledge Exchange. Dundee University: Design in Action. Available online at: http://www.designinaction.com/wp-content/uploads/2016/12/DiA-Final-Report.pdf (Accessed August 28, 2018).

[B30] Dixon-Woods M. Amalberti R. Goodman S. Bergman B. Glasziou P. (2011). Problems and promises of innovation: why healthcare needs to rethink its love/hate relationship with the new. BMJ Qual. Saf. 20 (Suppl. 1), 47–51. 10.1136/bmjqs.2010.046227 21450771 PMC3066840

[B31] Dovey J. Moreton S. Sparke S. Sharpe B. (2014). Curating Connectivity – REACT Working Paper No.3. Bristol: REACT Hub. Available online at: http://www.react-hub.org.uk/sites/default/files/publications/WORKING%20PAPER%203%20Curating%20Connectivity.pdf (Accessed August 28, 2018).

[B32] Dovey J. Pratt A. C. Moreton S. Virani T. Merkel J. Lansdowne J. (2016). Creative Hubs: Understanding the New Economy. London: British council. Available online at: https://creativeconomy.britishcouncil.org/media/uploads/files/HubsReport.pdf (Accessed August 28, 2018).

[B33] Dowlen R. Keady J. Milligan C. Swarbrick C. Ponsillo N. Geddes L.. (2017). The personal benefits of musicking for people living with dementia: a thematic synthesis of the qualitative literature. Arts Health. 10, 197–212. 10.1080/17533015.2017.1370718

[B34] Dowling A. (2015). The Dowling Review of Business-University Research Collaborations. London: BIS. Available online at: https://www.gov.uk/government/publications/business-university-research-collaborations-dowling-review-final-report (Accessed August 28, 2018).

[B35] East Dunbartonshire Council (2014). Co-production With People Living With Dementia. Glasgow: East Dunbartonshire Council. Available online at http://www.govint.org/fileadmin/user_upload/publications/Present_Project_Report_2014.pdf (Accessed August 28, 2018).

[B36] Equality Human Rights Commission (2011). Close to Home An Inquiry into Older People and Human Rights in Home Care. Equality and Human Rights Commission. Available online at: https://www.equalityhumanrights.com/en/publication-download/close-home-inquiry-older-people-and-human-rights-home-care (Accessed August 28, 2018).

[B37] Facer K. Enright B. (2016). Creating Living Knowledge: The Connected Communities Programme, Community-University Relationships and the Participatory Turn in the Production of Knowledge. Bristol: University of Bristol/AHRC. Available online at https://connected-communities.org/wp-content/uploads/2016/04/Creating-Living-Knowledge.Final_.pdf (Accessed August 28, 2018).

[B38] Fuller M. (2005). Media Ecologies: Materialist Energies in Art and Technoculture. Cambridge, MA: The MIT Press.

[B39] Gabriel M. Stanley I. Saunders T. (2017). Open Innovation in Health: A Guide to Transforming Healthcare Through Collaboration. London: Nesta. Available online at https://media.nesta.org.uk/documents/open_innovation_in_health_0.pdf (Accessed August 28, 2018).

[B40] Gould V. F. (2013). Reawakening the Mind: Evaluation of Arts4Dementia's London Arts Challenge in 2012. London: Arts4Dementia. Available online at https://www.museumsassociation.org/download?id=1062531 (Accessed August 28, 2018).

[B41] Gray K. Evans S. C. Griffiths A. Schneider J. (2017). Critical reflections on methodological challenge in arts and dementia evaluation and research. Dementia 17, 775–784. 10.1177/1471301217734478 28980477

[B42] Harris J. Rowley L. (2017). Working With Public Service Commissioners: A Quick Guide for the Arts and Cultural Sector. London: NCVO. Available online at https://knowhownonprofit.org/funding/commissioning/cultural-commissioning/resources-developed-by-the-cultural-commissioning-programme/WorkingWithCommissioners.pdf (Accessed August 28, 2018).

[B43] Herholz S. C. Herholz R. S. Herholz K. (2013). Non-pharmacological interventions and neuroplasticity in early stage Alzheimer's disease. Expert Rev. Neurother. 13, 1235–1245. 10.1586/14737175.2013.845086 24134650

[B44] Hippel E. V. (2005). Democratizing Innovation. Cambridge, MA; London: MIT Press. 10.7551/mitpress/2333.001.0001

[B45] Innovate Dementia (2019). Innovate Dementia: Innovative Care for Persons With Dementia. Available online at: http://www.innovatedementia.com/en (Accessed January 25, 2019).

[B46] International Futures Forum (2019). Three Horizons. iffpraxis.com. Available online at http://www.iffpraxis.com/three-horizons (Accessed January 25, 2019).

[B47] Kane M. Cook L. (2013). Dementia 2013: The Hidden Voice of Loneliness. London: Alzheimer's Society. Available online at: https://www.alzheimers.org.uk/sites/default/files/migrate/downloads/dementia_2013_the_hidden_voice_of_loneliness.pdf (Accessed August 28, 2018).

[B48] Khoury R. Patel K. Gold J. Hinds S. Grossberg G. T. (2017). Recent Progress in the pharmacotherapy of Alzheimer's Disease. Drugs Aging 34, 811–820. 10.1007/s40266-017-0499-x 29116600

[B49] Kontos P. Miller K. L. Kontos A. P. (2017). Relational citizenship: supporting embodied selfhood and relationality in dementia care. Soc Health Illn. 39, 182–198. 10.1111/1467-9566.12453 28177149

[B50] Lakey L. Chandaria K. Quince C. Kane M. Saunders T. (2012). Dementia 2012: A National Challenge. London: Alzheimer's Society, Available online at: https://www.alzheimers.org.uk/sites/default/files/migrate/downloads/alzheimers_society_dementia_2012-_full_report.pdf (Accessed August 28, 2018).

[B51] Lester R. K. Piore M. J. (2004). Innovation–the Missing Dimension. Cambridge, MA: Harvard University Press.

[B52] Malmberg K. Vaittinen I. (Eds) (2017). Living Lab Methodology Handbook. U4IoT Consortium. Available online at: https://u4iot.eu/pdf/U4IoT_LivingLabMethodology_Handbook.pdf (Accessed August 28, 2018).

[B53] Markusen A. Gadwa A. Barbour E. Beyers W. (2011). California's Arts and Cultural Ecology. San Francisco, CA: James Irvine Foundation. Available online at: http://annmarkusen.com/wp-content/uploads/2013/01/ca-arts-culture.pdf (Accessed February 12, 2019).

[B54] McNeil C. Hunter J. (2014). The Generation Strain Collective Solutions to Care in an Ageing Society. London: Institute for Public Policy Research. Available online at: https://www.ippr.org/files/publications/pdf/generation-strain_Apr2014.pdf (Accessed August 28, 2018).

[B55] Moreton S. Dovey D. (2013). Curating Collaboration: The Experience of Collaborative Innovation in REACT – REACT Working Paper No.2. Bristol: REACT Hub. Available online at: http://www.react-hub.org.uk/sites/default/files/publications/curating%20collaboration_final.pdf

[B56] Mukadam N. Livingston G. Rantell K. Rickman S. (2014). Diagnostic rates and treatment of dementia before and after launch of a national dementia policy: an observational study using English national databases. BMJ Open 4:e004119. 10.1136/bmjopen-2013-004119 24413352 PMC3902654

[B57] Pratt A. C. (2014). Putting Knowledge in (its) Place: Knowledge Exchange/Transfer and Clustering – Creativeworks London Working Paper No.5. London: City University London. Available online at: http://www.creativeworkslondon.org.uk/wp-content/uploads/2013/11/PWK-Working-Paper-5.pdf (Accessed August 28, 2018).

[B58] Prince M. Comas-Herrera A. Knapp M. Guerchet M. Karagiannidou M. (2016). World Alzheimer Report 2016: Summary Sheet. London: Alzheimer's Disease International, 2016. Available online at: https://www.alz.co.uk/research/WorldAlzheimerReport2016.pdf

[B59] Prince M. Knapp M. Guerchet M. McCrone P. Prina M. Comas-Herrera A.. (2014). Dementia UK Update 2014. London: Alzheimer's Society. Available online at: https://www.alzheimers.org.uk/sites/default/files/migrate/downloads/dementia_uk_update.pdf (Accessed August 28, 2018).

[B60] REACT (2016). REACT Report 2012–2016. Bristol: REACT Hub. Available online at: http://www.react-hub.org.uk/publications/react-report/ (Accessed August 28, 2018).

[B61] Robles A. G. Hirvikoski T. Schuu D. (2015). Introducing ENoLL and its Living Lab Community. Brussels: European Network of Living Labs. Available online at: https://issuu.com/enoll/docs/enoll-print (Accessed August 28, 2018).

[B62] Sargsyan G. Meijer G. Janssen W. van Buuren R. Husmann E. Ali-Vehmas T.. (2011). (Editor) Socio-Economic Impact of Open Service Innovation. Brussels: European Commission Information Society and Media. Available online at: https://ec.europa.eu/digital-single-market/en/news/socio-economic-impact-open-service-innovation-smart-20090077 (Accessed August 28, 2018).

[B63] Senior T. J. (2016). Connecting to Innovate: A Preliminary Report on the Achievements of the AHRC Knowledge Exchange Hubs for the Creative Economy. Bristol: University of the West of England. Available online at: http://www.react-hub.org.uk/sites/default/files/publications/Connecting-to-Innovate-Final.pdf (Accessed August 28, 2018).

[B64] Senior T. J. (2018a). Report 1: The Arts and Humanities in the Creative Economy: Core Learning from the AHRC Creative Economy Hubs Programme. Bristol: University of the West of England. Available online at: https://dementiaconnect.dcrc.org.uk/reports-media/ (Accessed August 28, 2018).

[B65] Senior T. J. (2018b). Report 2: A Framework for University-led Creative Economy Innovation: Core Learning from the AHRC Creative Economy Hubs programme. Bristol: University of the West of England. Available online at: https://dementiaconnect.dcrc.org.uk/reports-media/ (Accessed August 28, 2018).

[B66] Senior T. J. (2018c). Report 3: The Hub as Organisational Model in the Creative Economy: Core Learning from the AHRC Creative Economy Hubs Programme. Bristol: University of the West of England. Available online at: https://dementiaconnect.dcrc.org.uk/reports-media/ (Accessed August 28, 2018).

[B67] Senior T. J. Moreton S. Dovey J. (2015). The Arts and Humanities in the Internet of Things – REACT Working Paper. Bristol: REACT Hub. Available online at: http://www.react-hub.org.uk/sites/default/files/publications/AH%20in%20IoT.pdf (Accessed February 12, 2019)

[B68] Sharpe B. (2010). Economies of Life: Patterns of Health and Wealth. Charmouth: Triarchy Press.

[B69] Shiach M. Nakano D. Virani T. Poli K. (2017). Report on Creative Hubs and Urban Development Goals (UK/Brazil). London: Queen Mary University. Available online at: http://www.networkcentre.uk/wp-content/uploads/2018/02/Creative-Hubs-and-Urban-Development-Goals-UK-Brazil_Published.pdf (Accessed August 28, 2018).

[B70] The Creative Exchange (2016). The Creative Exchange Final Report to the AHRC. Lancaster: Lancaster University. Available online at: http://thecreativeexchange.org/activity/creative-exchange-final-report-published (Accessed August 28, 2018)

[B71] The King's Fund (2017). The NHS: How Providers are Regulated and Commissioned. London: The King's Fund. Available online at: https://www.kingsfund.org.uk/audio-video/how-new-nhs-structured (Accessed August 28, 2018).

[B72] Thomas G. E. C. Crutch S. J. Camic P. M. (2018). Measuring physiological responses to the arts in people with a dementia. Int. J. Psychophysiol. 123, 64–73. 10.1016/j.ijpsycho.2017.11.008 29158118

[B73] Transform Ageing (2018). Delivering a Better Experience for Later Life. London: Transform Ageing Consortium. Available online at: https://www.unltd.org.uk/uploads/general_uploads/TA_Innovation_Briefs_Brochure_180518u.pdf (Accessed August 28, 2018).

[B74] Tsekleves E. Bingley A. Escalante M. L. Gradinar A. (2015). Ageing Playfully Design Report. Lancaster: Lancaster University. Available online at: http://imagination.lancs.ac.uk/outcomes/Ageing_Playfully_Design_Report (Accessed August 28, 2018).

[B75] UWE Bristol (2018). Creative Producing. Courses.uwe.ac.uk. Available online at: https://courses.uwe.ac.uk/P3101/creative-producing (Accessed August 28, 2018)

[B76] Virani T. E. (2015a). Mechanisms of Collaboration Between Creative Small, Medium and Micro-Sized Enterprises and Higher Education Institutions: Reflections on the Creativeworks London CV Scheme (Creativework London Working Paper No. 4). London: Queen Mary University. Available online at: http://www.creativeworkslondon.org.uk/wp-content/uploads/2013/11/PWK-Working-Paper-4-SEO.pdf (Accessed August 28, 2018).

[B77] Virani T. E. (2015b). Do Voucher Schemes Matter in the Long Run? A Brief Comparison of Nesta's Creative Credits and Creativeworks London's CV schemes (Creativework London Working Paper No. 10). London: Queen Mary University. Available online at: http://www.creativeworkslondon.org.uk/wp-content/uploads/2013/11/Working-Paper-10.pdf (Accessed August 28, 2018).

[B78] Virani T. E. (2018). Dementia Connect: CVs Scheme Evaluation Report. Bristol: Dementia Connect. Available online at: https://dementiaconnect.dcrc.org.uk/reports-media/ (Accessed August 28, 2018).

[B79] Ward J. (2016). Social Prescribing at a Glance. North West England: A Scoping Report of Activity for the North West. Leeds: Health Education England. Available online at: https://www.hee.nhs.uk/sites/default/files/documents/Social%20Prescribing%20at%20a%20glance.pdf (Accessed August 28, 2018).

[B80] Wass S. Vimarlund V. (2016). Healthcare in the age of open innovation—a literature review. Health Inf. Manage. J. 45, 121–133. 10.1177/1833358316639458 27105481

[B81] West J. Salter A. Vanhaverbekec W. Chesbrough H. (2014). Open innovation: the next decade. Res. Policy 43, 805–811. 10.1016/j.respol.2014.03.001

[B82] WHO (2017). Global Action Plan on the Public Health Response to Dementia 2017-2025. Geneva: World Health Organisation. Available online at: https://www.who.int/mental_health/neurology/dementia/action_plan_2017_2025/en/ (Accessed August 28, 2018).

[B83] Windle G. (2018). Exploring the theoretical foundations of visual art programmes for people living with dementia. Dementia 17, 702–727. 10.1177/1471301217726613 28914090 PMC6068961

[B84] Windle G. Gregory S. Newman A. Goulding A. O'Brien D. Parkinson C. (2014). Understanding the impact of visual arts interventions for people living with dementia: a realist review protocol. Syst. Rev. 3:91. 10.1186/2046-4053-3-91 25128286 PMC4141269

[B85] Windle G. Joling K. J. Howson-Griffiths T. Woods B. (2018). The impact of a visual arts program on quality of life, communication, and well-being of people living with dementia: a mixed-methods longitudinal investigation. Int. Psychogeriatr. 30, 409–423. 10.1017/S1041610217002162 29113610

[B86] Windle G. Newman A. Burholt V. Woods B. O'Brien D. Baber M.. (2016). Dementia and Imagination: a mixed-methods protocol for arts and science research. BMJ Open 6, 1–11. 10.1136/bmjopen-2016-011634 27807080 PMC5129039

[B87] Woods L. Pendleton J. Smith G. M. Parker P. (2015). Working Collaboratively to Support People Living with Dementia. Liverpool: Liverpool John Moores University. http://researchonline.ljmu.ac.uk/4149/ (Accessed August 28, 2018).

[B88] Woods L. Smith G. M. Pendleton J. Parker A. D. (2013). Innovate Dementia Baseline Report: Shaping the Future for People Living with Dementia. Liverpool: Liverpool John Moores University. Available online at: http://researchonline.ljmu.ac.uk/4152/1/Innovate%20Dementia%20Baseline%20report.pdf (Accessed August 28, 2018).

[B89] Woods M. Marra M. Coulson S. (2015). Design in Action Knowledge Exchange Process Model. Dundee: University of Dundee. Available online at: https://discovery.dundee.ac.uk/ws/portalfiles/portal/7754918/Woods_Marra_Coulson_2015.pdf (Accessed August 28, 2018).

[B90] Woodward M. Arthur A. Darlington N. Buckner S. Killett A. Thurman J.. (2018). The place for dementia-friendly communities in England and its relationship with epidemiological need. Int. J. Geriatr. Psychiatry 34, 67–71. 10.1002/gps.4987 30248208 PMC6585629

[B91] Wootten J. Chandaria K. Graty C. Newby C. Harkin S. Gascoigne A. (2016). Becoming a Dementia-Friendly Retailer: A Practical Guide. London: Alzheimer's Society. Available online at: https://www.alzheimers.org.uk/sites/default/files/migrate/downloads/dementia_friendly_retail_guide.pdf (Accessed August 28, 2018).

[B92] Young R. Camic P. M. Tischler V. (2016). The impact of community-based arts and health interventions on cognition in people with dementia: a systematic literature review. Aging Ment. Health 20, 337–351. 10.1080/13607863.2015.1011080 25683767

[B93] Zeilig H. West J. van der Byl Williams M. (2018). Co-creativity: possibilities for using the arts with people with a dementia. Q. Ageing Older Adults 19, 135–145. 10.1108/QAOA-02-2018-0008

